# Genome-Wide Identification, Drought-Responsive Expression, and EAR-Mediated Regulatory Network Construction of *TOPLESS* Genes in *Populus ussuriensis Kom.*

**DOI:** 10.3390/plants14203213

**Published:** 2025-10-19

**Authors:** Wanxin Li, Qianqian Liu, Jingru Ren, Zihan Fan, Tabeer Gulfam, Zhongzheng Ma, Jingli Yang

**Affiliations:** State Key Laboratory of Forest Genetics and Tree Breeding, Northeast Forestry University, Harbin 150040, China; li8269681@163.com (W.L.); 13069825661@139.com (Q.L.); 15931322806@163.com (J.R.); fanshi26528@163.com (Z.F.); tabeergulfam11@gmail.com (T.G.); 18553757642@163.com (Z.M.)

**Keywords:** *Populus ussuriensis*, Topless gene family, drought stress, transcriptional repression, EAR motif

## Abstract

Drought stress significantly impairs plant growth and productivity, which triggers complex adaptive responses mediated by diverse gene families. Among these, the TOPLESS (TPL)/TPL-related (TPR) family of transcriptional corepressors plays a crucial role by recruiting epigenetic modifiers through interactions with EAR motif-containing proteins. However, genome-wide studies of this corepressor family and its associated regulatory networks with EAR motif-containing repressors remain limited. This study aimed to characterize the TPL/TPR transcriptional corepressor family in *Populus ussuriensis Kom*., elucidate their regulatory networks with EAR motif-containing repressors, and validate their functional roles in drought stress adaptation. To this end, we identified 21 *TPL/TPR* genes in *P. ussuriensis* (*PuTPLs*), classified them into five subfamilies, and found they are evolutionarily conserved with *Arabidopsis thaliana* and *Populus trichocarpa*, harboring characteristic CTLH and WD40 domains. Given that TPL/TPR proteins are recruited by transcription factors containing repression motifs, we constructed a putative TPL/TP*R*-EAR motif interaction network representing a core paradigm of negative regulation. Expression profiling under drought stress showed significant upregulation of most *PuTPLs* in a tissue-specific and temporal manner. Functional validation using transgenic *P. ussuriensis* lines overexpressing five *PuTPLs* demonstrated enhanced drought tolerance, evidenced by reduced electrolyte leakage and malondialdehyde content and increased proline accumulation. Our study provides the first comprehensive genome-wide analysis of the TPL/TPR family in *P. ussuriensis*, establishes a core EAR-mediated negative regulatory network, and validates the critical role of these genes in drought stress adaptation, providing valuable resources for future mechanistic research and breeding of stress-resistant trees.

## 1. Introduction

Drought is a major abiotic stress that severely restricts plant growth, development, and agricultural productivity worldwide. Under water-deficient conditions, plants undergo various physiological and biochemical disturbances, resulting in reduced biomass accumulation, inhibited growth, cellular dehydration, and declined photosynthetic efficiency [1,2,3]. To cope with drought stress, plants have evolved diverse adaptive mechanisms, such as remodeling root architecture [4,5,6], promoting stomatal closure [7], modifying xylem structure [8], and activating multiple signaling pathways [9,10]. Transcriptional regulation serves a central mechanism underlying drought responses. Numerous transcription factors (TFs)—including ARF, NAC, and bZIP families—orchestrate adaptive gene expression programs under drought conditions [11,12,13]. These TFs are broadly categorized into transcriptional activators and repressors [14]. In recent years, the molecular mechanisms by which transcriptional repressors recruit corepressor complexes have attracted increasing research interest.

Unlike sequence-specific TFs, transcriptional corepressors do not bind DNA directly. Instead, they contain modular protein–interaction surfaces that enable assembly into repressive complexes with TFs transcriptional corepressors and chromatin modifiers [15]. Transcriptional repressors suppress gene expression through two primary modes: passive competition with activators for binding sites, or active recruitment of corepressors. Many repressors carry short repression domains (RDs) that mediate corepressor recruitment [16]. The most widespread RD is the EAR motif (LxLxL/DLNxxP), located in N- or C-terminal regions of certain TFs, which recruits corepressors to suppress gene expression [15,16]. In plants, transcriptional corepression is mediated by the Groucho/Tup1 family, which includes the TPL/TP*R* family, *LEUNIG* (*LUG*), *LEUNIG HOMOLOG* (*LUH*), and *WUSCHEL-INTERACTING PROTEINS* (*WSIPs*) [17]. These corepressors form multiprotein complexes that recruit epigenetic modifiers such as histone deacetylases (*HDACs*) to modulate chromatin state and repress transcription. Together, they regulate a wide range of biological processes, including growth, development, morphogenesis, and stress adaptation [18,19,20,21,22]. Among these, the TPL/TPR family acts as a central regulatory hub. TPL/TPR proteins recognize the EAR motif in repressor TFs—either directly or through adaptors [23]—and recruit *HDAC*-containing complexes. This leads to chromatin compaction via histone deacetylation (e.g., H3K27), methylation (H3K27me3), and nucleosome remodeling, ultimately suppressing transcriptional initiation [24].

Drought triggers multi-layered transcriptional networks. Within these networks, repressors recruit corepressors and, through their interaction, jointly control the expression of target genes, thereby balancing plant growth and stress adaptation. Among these, the highly conserved TPL/TPR family is a representative plant corepressor. Although first identified in embryonic and organ development [25,26,27], it is now recognized as a key regulator of stress adaptation. It interacts with EAR motif-containing drought-responsive TFs, including *ERF/AP2* and *MYB* family members, and recruits *HDACs* to modify chromatin [28,29]. These mechanisms modulate hormone signaling and suppress drought-responsive genes, enabling dynamic phenotypic adjustment and improved fitness under water scarcity.

*Populus ussuriensis Kom.*, a member of the genus Populus within the Salicaceae family, is an ecologically adaptable, fast-growing tree species with substantial economic value [30]. In this study, a comprehensive identification and characterization of all *TPL/TPR* genes in *P. ussuriensis* (*PuTPLs*) was performed. We constructed a regulatory network linking *PuTPLs* with EAR motif-containing TFs, supported by experimental validation. We further analyzed *PuTPLs* expression under drought stress and confirmed drought-responsive functions using transient transformation assays. Our findings provide a foundation for elucidating the regulatory networks between transcriptional coregulators and repressive TFs, offering actionable genetic targets and a scalable framework for breeding drought-resistant trees. This work significantly advances our mechanistic understanding of corepressor functions and contributes to global efforts to mitigate forest productivity losses under climate change.

## 2. Results

### 2.1. Identification and Phylogenetic Analysis of the TPL/TPR Genes in P. ussuriensis

**Figure 1:**
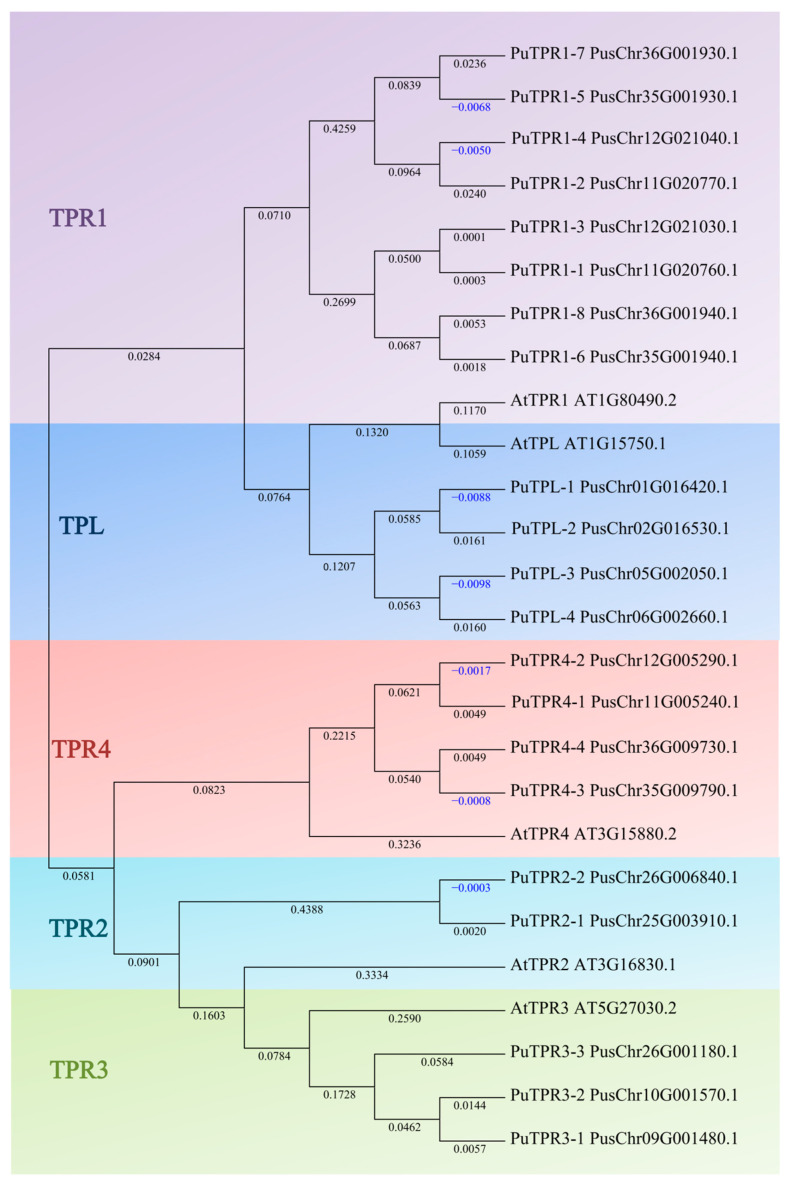
Phylogenetic analysis of *TPL/TPR* genes from *P. ussuriensis* and *A. thaliana*. Different gene subfamilies are color-coded: *PuTPL* (blue), *PuTPR1* (purple), *PuTPR2* (cyan), *PuTPR3* (green), and *PuTPR4* (red). Numbers on the branches indicate branch lengths.

To identify *TPL/TPR* genes in *P. ussuriensis*, we performed a BLASTN search using *TPL/TPR* sequences from the model plant *Arabidopsis thaliana* as queries. This search identified 21 putative *TPL/TPR* homologs in the *P. ussuriensis* genome. To assess evolutionary conservation and infer phylogenetic relationships, we constructed a neighbor-joining (NJ) phylogenetic tree using MEGA12 with the full-length coding sequences of the 21 *PuTPLs* and 5 *AtTPLs* (*TPL/TPR* genes in *A. thaliana*). To better resolve functional diversity and investigate gene duplication events, the 21 *PuTPLs* were classified into five subfamilies based on the phylogenetic topology (Figure 1): *PuTPL* (*Protein Topless*), *PuTPR1* (*Topless-related protein 1*), *PuTPR2* (*Topless-related protein 2*), *PuTPR3* (*Topless-related protein 3*), and *PuTPR4* (*Topless-related protein 4*). The classification yielded four *PuTPL* genes, eight *PuTPR1* genes, two *PuTPR2* genes, three *PuTPR3* genes, and four *PuTPR4* genes. These results suggest that although *TPL/TPR* genes retain conserved core functions, lineage-specific functional divergence has occurred between *A. thaliana* and *P. ussuriensis*, which may contribute to species-specific adaptive strategies in response to environmental stresses.

### 2.2. Physicochemical Properties and Secondary Structures of TPL/TPR Proteins in P. ussuriensis

**Figure 2:**
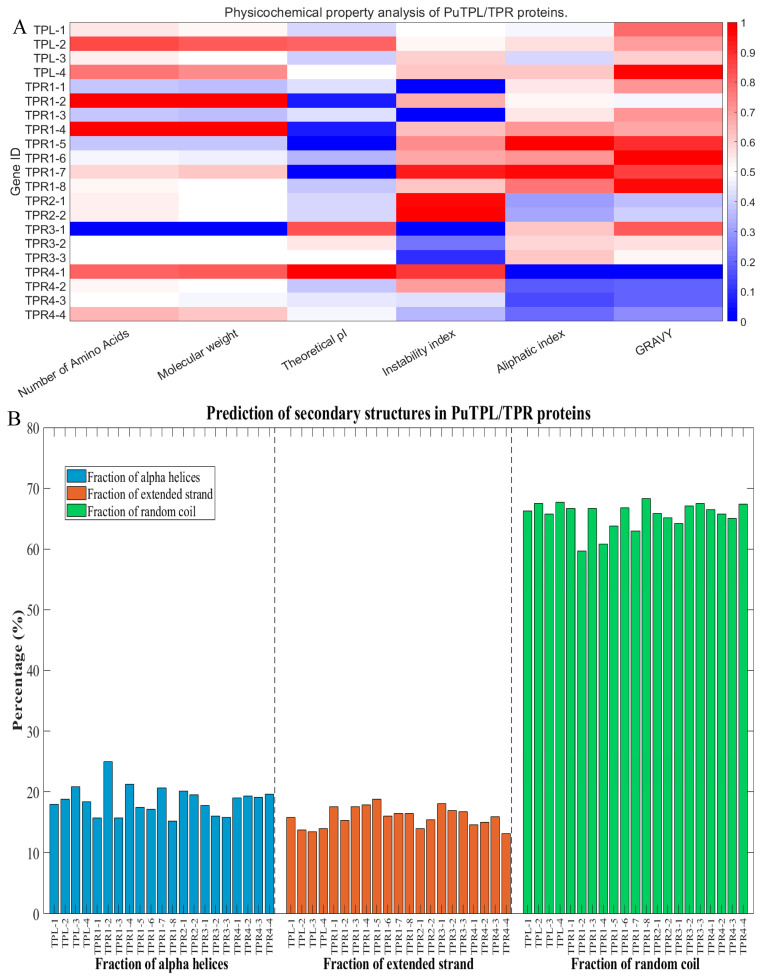
Physicochemical properties and secondary structures of TPL/TPR proteins in *P. ussuriensis*. (**A**) Heatmap displaying key physicochemical properties: molecular weight (kDa), theoretical pI, instability index, aliphatic index, and GRAVY. (**B**) Predicted secondary structure composition of each TPL/TPR protein, showing the proportion of α-helices, β-sheets, and random coils.

Bioinformatic analysis revealed distinct physicochemical characteristics among PuTPL/TPR proteins (Figure 2A). The proteins ranged from 963 to 1285 amino acids in length, with molecular weights between 106.8 and 142.3 kDa. Isoelectric points (pI) varied from acidic to alkaline; four proteins exhibiting a basic pI (>7) and seventeen were acidic pI (<7). Based on instability index values, nine proteins were classified as stable (<40) and the remainder as unstable. The average aliphatic index was 82.58, suggesting generally high thermostability. All proteins were hydrophilic, as indicated by negative grand average of hydropathicity (GRAVY) values (Supplementary Table S1).

As shown in Figure 2B, secondary structure prediction revealed consistent compositional features across all PuTPL/TPR proteins. Alpha-helix content ranged from 15.22% to 24.98%, primarily due to conserved helix-turn-helix (HTH) motifs localized within CTLH domains. Extended strand structures, which are predominant in WD40 repeat regions, varied from 13.08% to 18.80%, consistent with their role in maintaining structural stability. All proteins exhibited a high proportion of random coil conformations (59.69–68.32%), implicating substantial structural flexibility that may facilitate dynamic protein–protein interactions during transcriptional repression (Supplementary Table S2).

### 2.3. Gene Structure, Conserved Domains, and Cis-Regulatory Elements

**Figure 3:**
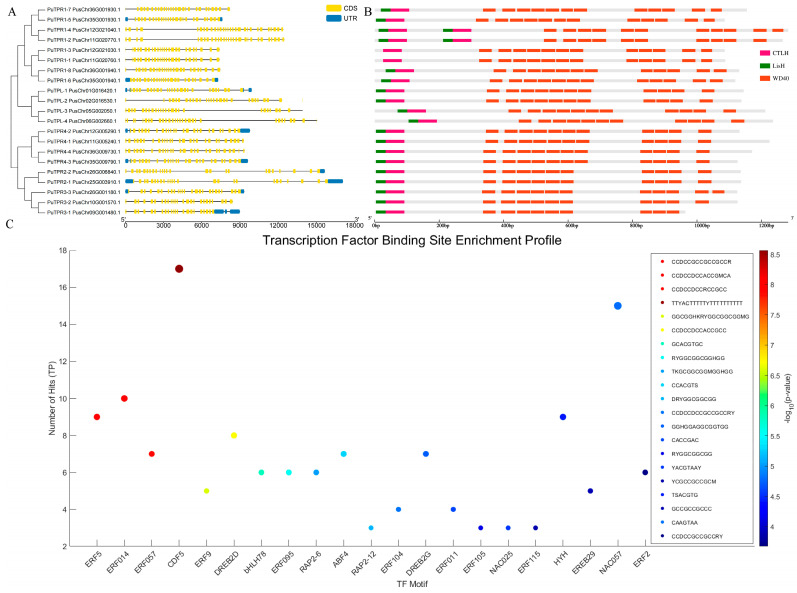
Gene structure, conserved domains, and cis-regulatory elements of TPL/TPR family members. (**A**) Exon–intron structures of 21 *PuTPL*s. CDS (yellow), introns (black lines), and UTRs (blue) are shown. (**B**) Conserved protein domains. Domains are color-coded: CTLH (pink), LisH (green), and WD40 (Orange). (**C**) Enrichment of transcription factor (TF) binding sites. The plot shows the number of binding sites (hits) for each TF, colored by the statistical significance of enrichment. The corresponding DNA binding motif is shown for each TF.

In contrast to most genes in *P. ussuriensis*, the *TPL/TPR* genes exhibit a compact organization with high exon density, characterized by the presence of multiple short exons ranging from 80 to 300 bp in all members (Figure 3A). This gene structure is conserved in their homologs from *P. trichocarpa* (Supplementary Figure S1A). As shown in Figure 3B, all PuTPL/TPR proteins contain the fundamental N-terminal CTLH domain and multiple C-terminal WD40 repeats, which are hallmark features of the TPL/TPR family. Notably, *PuTPR1-1* and *PuTPR1-3* lack the conserved LisH motif, whereas *PuTPR1-2* and *PuTPR1-4* possess duplicate sets of both LisH and CTLH domains. The overall protein domain architecture is largely conserved across species (Supplementary Figure S1B), although the increased number of WD40 repeats and the duplication of LisH/CTLH modules in certain *PuTPLs* may indicate functional diversification within this family.

Our *cis*-regulatory element enrichment analysis identified 21 drought-associated motifs, corresponding to transcription factors from multiple families implicated in stress and hormonal adaptation. These include not only core abiotic stress regulators from the AP2/ERF (e.g., DREB2D, DREB2G), NAC (NAC025), and bZIP (ABF4) families, but also key components of hormone signaling pathways, particularly those responsive to abscisic acid (ABF4) and ethylene (ERF5, ERF014, ERF057). The significant overrepresentation of these stress- and hormone-responsive regulators strongly supports the role of PuTPLs as co-repressors in integrative drought-responsive transcriptional networks (Figure 3C).

### 2.4. Chromosomal Localization and Synteny Analysis of PuTPLs

**Figure 4:**
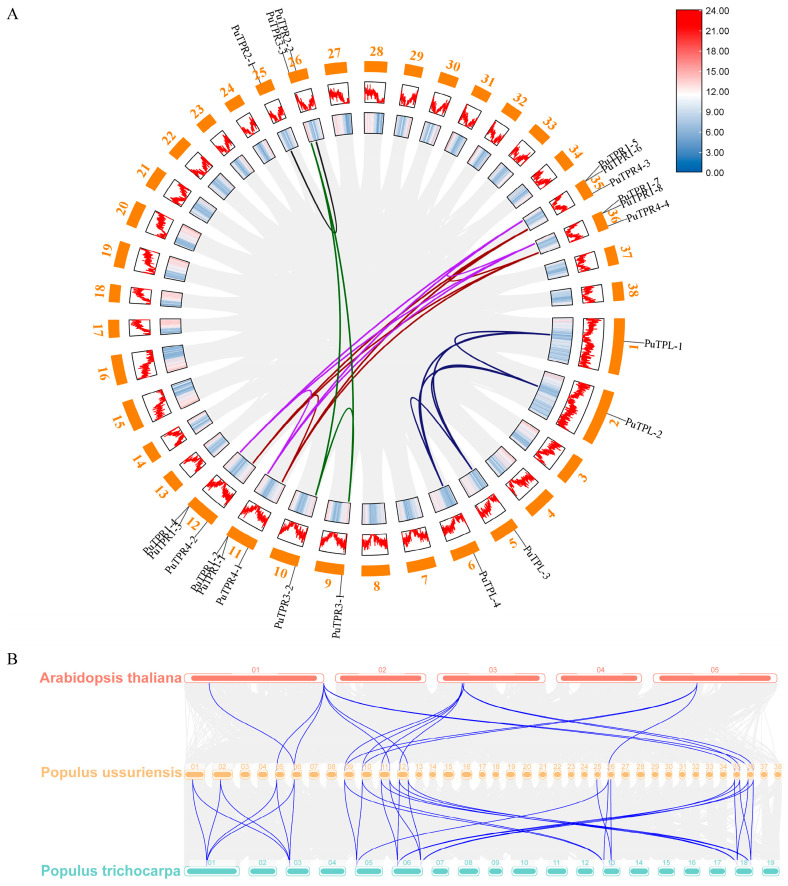
Chromosomal locations and synteny of *PuTPLs*. (**A**) Intra-genomic collinearity of *PuTPLs*. Paralogous gene pairs are connected by colored lines. The positions of *PuTPLs* are indicated on the chromosomes, which are arranged in a circle. Gene density is shown as a heatmap (inner ring) and a line graph (outer ring). (**B**) Synteny relationships of TPL/TPR genes across *P. ussuriensis*, *A. thaliana*, and *P. trichocarpa*. Orthologous gene pairs are linked by curved lines. Chromosomes from each species are colored differently.

As shown in Figure 4A, cytogenetic mapping placed the four *PuTPL* genes on chromosomes 1, 2, 5, and 6. Although chromosomes 1/2 and 5/6 are homologous pairs, the *PuTPL* loci showed a non-syntenic arrangement between chromosomes 5 and 6. The eight *PuTPR1* genes were distributed as two adjacent pairs on homologous chromosomes 11/12 and 35/36. The two *PuTPR2* genes were located on homologous chromosomes 25 and 26, while the three *PuTPR3* genes were found on chromosomes 9, 10, and 26. A large deletion on chromosome 25 relative to chromosome 26 likely caused the loss of the *PuTPR3* locus and a translocation of *PuTPR2* on chromosome 25. All four *PuTPR4* genes resided on homologous pairs 11/12 and 35/36 (Supplementary Figure S2).

Collinearity analysis revealed 22 paralogous segmental duplication events, including those between homologous chromosomes, which corroborates the inferred phylogenetic relationships. The absence of tandem duplications suggests evolutionary divergence among adjacent *PuTPR*1 genes (Figure 4A). *A. thaliana* (At) contains fewer *TPL/TPR* genes, and no orthologs of *PuTPL-1*, *PuTPL-2*, or *PuTPR2* were detected in this species. Orthology analysis supports the phylogenetic topology wherein *PuTPL* and *PuTPR1* genes cluster into one clade, while the other three subfamilies form a distinct evolutionary branch. Comparative genomic analysis further demonstrated high homology between *Populus trichocarpa* (Ptr) and *P. ussuriensis* genes (Figure 4B)

### 2.5. The TPL/TPR Co-Repressor Interaction Network Mediated by EAR Motifs

**Figure 5:**
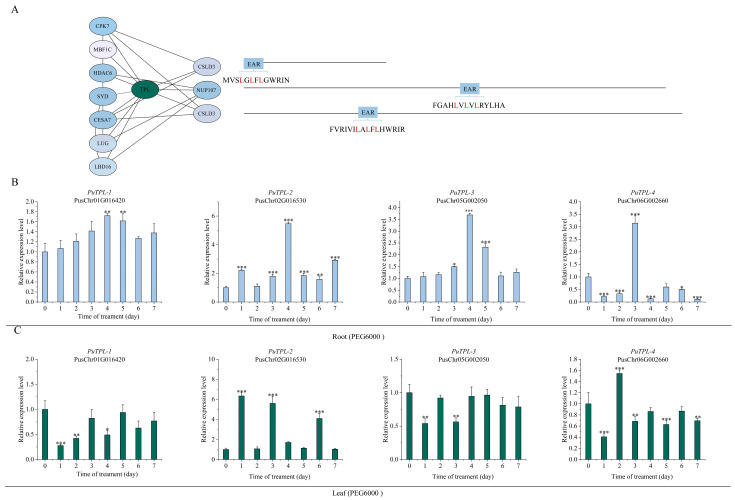
Interaction network and drought stress expression analysis of PuTPL. (**A**) Interaction network of PuTPL. Node color intensity represents the confidence score of the interactions, with darker shades indicating higher confidence. Interacting proteins are categorized based on the presence (right) or absence (left) of an EAR motif. The specific EAR motif sequences for relevant proteins are displayed. (**B**) Relative expression levels of *PuTPL* genes in roots under drought stress at various time point. (**C**) Relative expression levels of *PuTPL* genes in leaves under drought stress at various time points. For (**B**,**C**): Expression was normalized to *PuActin* as an internal reference. Data are presented as the mean ± SD of three biological replicates. Statistical significance was determined by one-way ANOVA (Tukey’s test): * *p* ≤ 0.05, ** *p* ≤ 0.01, *** *p* ≤ 0.001.

**Figure 6:**
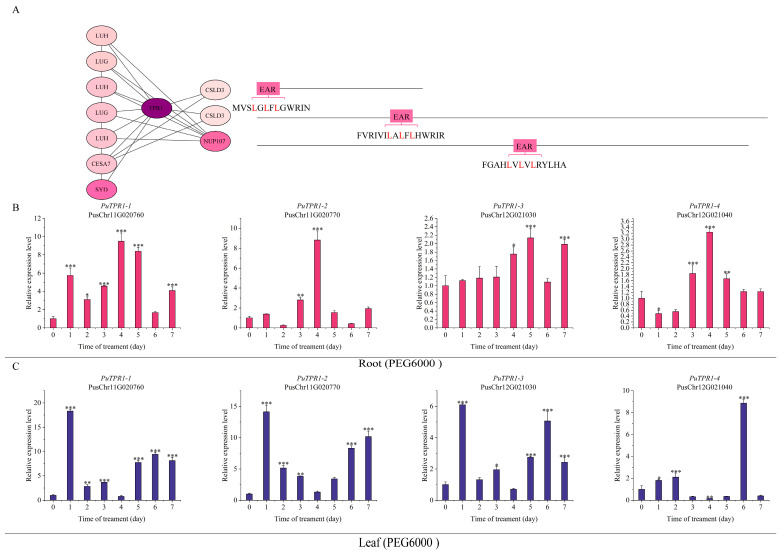
Interaction network and drought stress expression analysis of PuTPR1. (**A**) Interaction network of PuTPR1. Node color intensity indicates interaction confidence, with darker shades indicating higher confidence. Interacting proteins are categorized based on the presence (right) or absence (left) of an EAR motif. The specific EAR motif sequences for relevant proteins are shown. (**B**) Relative expression levels of *PuTPR1* in roots at different time points under drought stress. (**C**) Relative expression levels of *PuTPR1* in leaves at different time points under drought stress. For (**B**,**C**): Gene expression was normalized to *PuActin* used as an internal reference. Data are presented as the mean ± SD (*n* = 3 biological replicates). Statistical significance was determined by one-way ANOVA (Tukey’s test): * *p* ≤ 0.05, **p ≤ 0.01, *** *p* ≤ 0.001.

**Figure 7:**
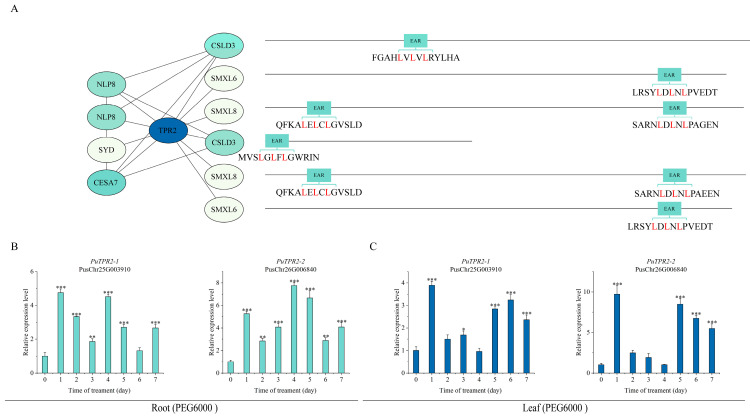
Interaction network and drought stress expression analysis of PuTPR2. (**A**) Interaction network of PuTPR2. Node color gradient indicates the confidence score of interactions. Interacting proteins are grouped based on the presence (right) or absence (left) of an EAR motif. The specific EAR motif sequences for relevant proteins are shown. (**B**) Relative expression levels of *PuTPR2* in roots at different time points under drought stress. (**C**) Relative expression levels of *PuTPR2* in leaves at different time points under drought stress. For (**B**,**C**): Expression was normalized to PuActin as an internal reference. Data are presented as mean ± SD of three biological replicates. Statistical significance was determined by one-way ANOVA (Tukey’s test): * *p* ≤ 0.05, ** *p* ≤ 0.01, *** *p* ≤ 0.001 (Tukey’s test).

**Figure 8:**
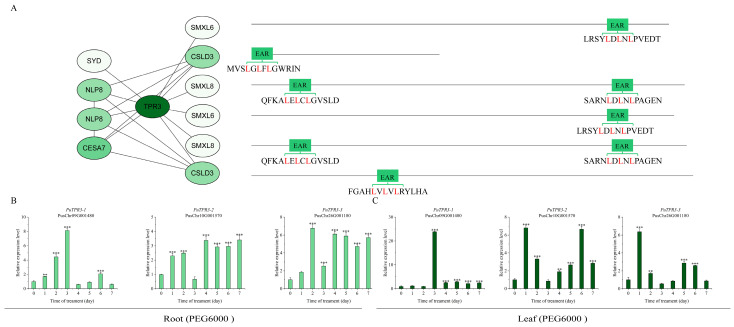
Interaction network and drought stress expression analysis of PuTPR3. (**A**) Interaction network of PuTPR3. Interaction confidence scores are visualized by node color intensity. Interacting proteins are grouped based on the presence (right) or absence (left) of an EAR motif. The specific EAR motif sequences for relevant proteins are shown. (**B**) Relative expression levels of *PuTPR3* in roots at different time points under drought stress. (**C**) Relative expression levels of *PuTPR3* in leaves at different time points under drought stress. For (**B**,**C**): Gene expression was normalized to *PuActin* as an internal reference. Data are presented as the mean ± SD of three biological replicates. Statistical significance between time points was determined by one-way ANOVA (Tukey’s test): ** *p* ≤ 0.01, *** *p* ≤ 0.001.

**Figure 9:**
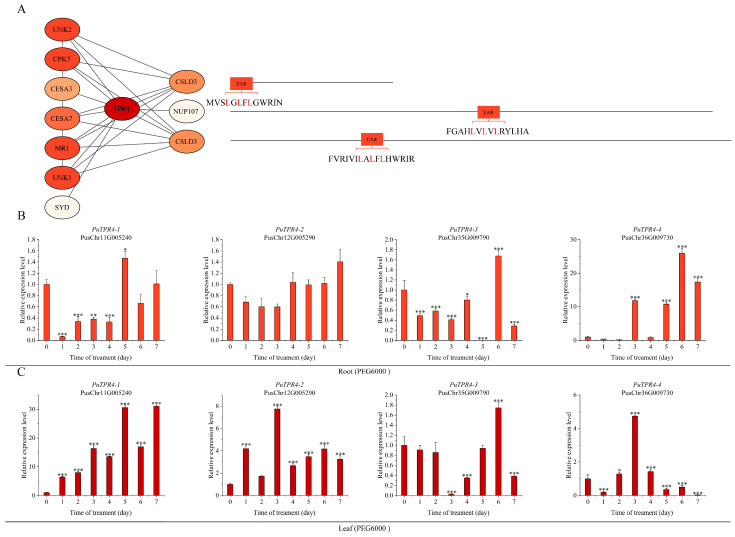
Interaction network and drought stress expression analysis of PuTPR4. (**A**) Interaction network of PuTPR4. Node color intensity represents the confidence score of interactions, with darker shades indicating higher confidence. Interacting proteins are grouped based on the presence (right) or absence (left) of an EAR motif. The specific EAR motif sequences for relevant proteins are shown. (**B**) Relative expression levels of *PuTPR4* in roots at different time points under drought stress. (**C**) Relative expression levels of PuTPR4 in leaves at different time points under drought stress. For (**B**,**C**): Expression was normalized to *PuActin* as an internal reference. Data are presented as mean ± SD of three biological replicates. Statistical significance between time points was determined by one-way ANOVA (Tukey’s test): * *p* ≤ 0.05, ** *p* ≤ 0.01, *** *p* ≤ 0.001.

We constructed phylogenetic trees for *TPL*, *TPR1*, *TPR2*, *TPR3*, and *TPR4* genes from *P. ussuriensis*, *P. trichocarpa*, and *A. thaliana* using MEGA12 (Supplementary Figures S4–S8). Based on the phylogenetic analysis, we selected five representative *P. trichocarpa* genes for interaction network screening using STRING database. The resulting networks were visualized and analyzed with Cytoscape software v3.10.3, and protein IDs were mapped to their corresponding genes. Subsequently, based on expression pattern analyses (Figure 5, Figure 6, Figure 7, Figure 8 and Figure 9), we cloned the closest *P. ussuriensis* homologs of these candidate genes to experimentally validate the predicted interaction networks.

PuTPL demonstrated interactions with several key regulators, including PuCPK7 (Calcium-Dependent Protein Kinase 7), PuSYD (SPLAYED), PuLUG (LEUNIG), PuHDAC6 (Histone Deacetylase 6), PuLBD16 (LATERAL ORGAN BOUNDARIES DOMAIN 16), PuMBF1C (Multiprotein Bridging Factor 1C) and PuCESA7 (Cellulose Synthase A7). Notably, two interacting partners PuCSLD3 (Cellulose Synthase-Like D3) and PuNUP107 (Nucleoporin 107) contained canonical EAR repression motifs (LxLxL or DLNxxP) (Figure 5A). Screening indicated that PuTPR1 bound to PuCSLD3 and PuNUP107, both containing EAR motifs, as well as to PuLUH (LEUNIG_HOMOLOG). The interaction profile of PuTPR1 showed significant similarity to that of PuTPL, suggesting functional conservation between these corepressors (Figure 6A). PuTPR2 interacted with multiple partners, including the transcription factors PuNLP8 (NIN-LIKE PROTEIN 8) and PuSYD, the cellulose synthase PuCESA7, and three additional EAR motif-containing proteins: PuCSLD3, PuSMXL6 (SUPPRESSOR OF MAX2-LIKE 6), and PuSMXL8 (SUPPRESSOR OF MAX2-LIKE 8) (Figure 7A). PuTPR3 shared a similar interaction profile with PuTPR2 (Figure 8A), whereas PuTPR4 associated with EAR motif-containing proteins similarly to PuTPR1. Additionally, PuTPR4 exhibited novel interactions with PuMRI (MYB-RELATED INTERACTOR) and two previously uncharacterized proteins (Figure 9A).

As shown in Figure 5, Figure 6, Figure 7, Figure 8 and Figure 9, TPL/TPR proteins from each subfamily can interact with EAR motif-containing proteins. These interactors can be broadly categorized into two classes: transcription factors that actively recruit TPL/TPR corepressors to repress downstream gene expression, and non-transcriptional functional proteins that harbor a chimeric EAR motif. The latter facilitate TPL/TPR recruitment via their EAR domain, localizing the corepressor complex near target genes to enable transcriptional regulation.

**Figure 10:**
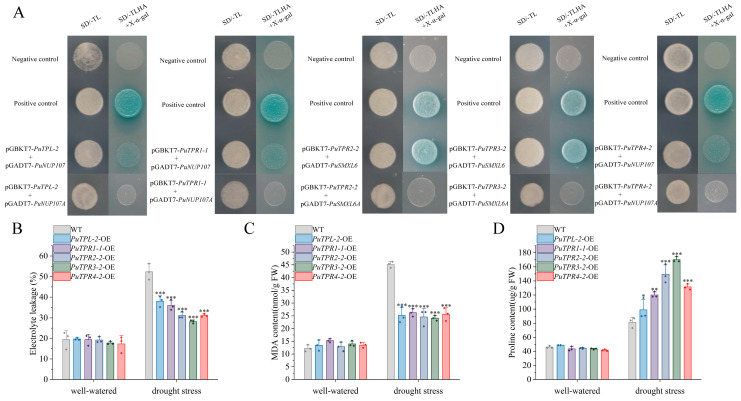
Validation of protein–protein interactions and physiological indices of drought tolerance. (**A**) Y2H assay validating interactions between PuNUP107 and PuTPL-2/PuTPR1-1/PuTPR4-2, and between PuSMXL6 and PuTPR2-2/PuTPR3-2. PuNUP107A and PuSMXL6A are EAR motif-mutant versions of PuNUP107 and PuSMXL6, respectively. Yeast cells were grown on SD/-Trp-Leu-His-Ade (SD/-TLHA) selection medium and assayed for X-α-gal activity. Blue color indicates a positive interaction. (**B**) Electrolyte leakage in wild-type (WT) and transient *PuTPLs-OE* lines under drought stress. (**C**) Malondialdehyde (MDA) content in WT and *PuTPLs-OE* lines under drought stress. (**D**) Proline (Pro) content in WT and PuTPLs OE lines under drought stress. For (**B**–**D**): Data are presented as mean ± SD (n = 3 biological replicates). Statistical significance between the OE lines and the WT under drought stress was determined by one-way ANOVA (Tukey’s test): * *p* ≤ 0.05, ** *p* ≤ 0.01, *** *p* ≤ 0.001.

To determine whether TPL/TPR–EAR interactions depend on a transcription factor context, we selected the EAR-containing nucleoporin PuNUP107 and the transcription factor PuSMXL6 for experimental validation. Yeast two-hybrid (Y2H) assays confirmed that TPL/TPR proteins bind the EAR motif directly, independent of transcription factor partners. Specifically, weak interactions were detected between PuNUP107 and PuTPL-2, PuTPR1-1, and PuTPR4-2, whereas PuTPR2-2 and PuTPR3-2 showed strong binding to PuSMXL6 (Figure 10A).

To validate the critical role of the EAR motif in mediating protein–protein interactions, site-directed mutagenesis was performed to construct two EAR motif mutants: PuNUP107A (with the conserved LVLVL sequence substituted to AVAVA) and PuSMXL6A (with the LDLNL motif mutated to ADANA). These mutations specifically targeted the core hydrophobic residues (Leu/Val) within the EAR motif. Yeast two-hybrid (Y2H) assays were then conducted to compare the interaction patterns between unmutated and mutant proteins with TPL/TPR family members.

Consistent with our hypothesis, the Y2H results revealed a complete loss of interaction between PuNUP107A and its previously identified partners (PuTPL-1, PuTPR1-2, PuTPR4-2), while the unmutated PuNUP107 showed strong binding activity as indicated by blue colony coloration on X-α-gal-containing medium. Similarly, PuSMXL6A failed to interact with PuTPR2-2 and PuTPR3-2, in contrast to the distinct positive signals observed for the unmutated PuSMXL6. These findings provide direct experimental evidence that the integrity of the EAR motif is indispensable for mediating the physical interactions between these proteins and TPL/TPR family members, supporting our bioinformatics prediction that EAR motifs serve as key binding interfaces in these regulatory interactions (Figure 10A).

### 2.6. Expression Patterns of PuTPLs Under Drought Stress

A systematic 7-day drought stress time-course experiment was conducted to examine the expression dynamics of *PuTPLs* in root and leaf tissues. The analysis revealed significant upregulation in 85% of the examined family members in roots under drought stress (*p* < 0.05, fold-change > 2). Among these responsive genes, 57% exhibited a characteristic pattern of initial induction followed by gradual decline, with expression peaks occurring during both early (1–2 days) and middle (3–5 days) drought stages. In leaf tissues, the response profile differed: 33% of genes were downregulated, while 61% of all genes showed stress-responsive upregulation. The genes exhibited tissue-specific expression patterns, with expression amplitude varying substantially between roots and leaves—reaching differences of up to 30-fold for *PuTPR4*—indicating tissue-specific regulatory mechanisms. The variation in peak expression timing and fold-change levels across genes is consistent with functional diversification rather than redundancy (Figure 5, Figure 6, Figure 7, Figure 8 and Figure 9).

Analysis revealed distinct expression patterns among different subfamilies: downregulation was observed in a subset of *PuTPL*, *PuTPR1*, *and PuTPR4* members (Supplementary Figure S3), suggesting specialized regulatory mechanisms in stress responses. In contrast, no downregulated genes were detected in the *PuTPR2* and *PuTPR3* subfamilies (Figure 5, Figure 6, Figure 7, Figure 8 and Figure 9), which is consistent with their phylogenetic divergence into independent clades (Figure 1). These differential expression patterns among TPL/TPR subfamilies support substantial functional diversification within this gene family during drought adaptation.

Expression analyses demonstrated that *PuTPR1-1* and *PuTPR1-3*, which lack the LisH domain, exhibited drought-responsive patterns like other family members (Figure 3 and Figure 6), suggesting that the LisH domain is not essential for mediating drought stress responses. In contrast, *PuTPR1-2* and *PuTPR1-4*, both contain dual LisH and CTLH domains, displayed distinct response characteristics. In roots, these genes showed no early response (0–3 days) to drought but were significantly upregulated after day 3, peaking at day 4. In leaves, *PuTPR1-2* was upregulated during both early and intermediate drought stages, whereas *PuTPR1-4* showed transient downregulation. The unique expression patterns of these dual-domain genes suggest that the CTLH domain may play an important role in drought responsiveness. Furthermore, the regulatory divergence between *PuTPR1-2* and *PuTPR1-4* implies the involvement of additional gene-specific factors in fine-tuning stress adaptation.

### 2.7. Physiological Analysis of Drought Tolerance in PuTPLs-Overexpressing (OE) Lines

We generated transient overexpression lines for five *PuTPLs*-OE lines and subjected them to drought stress simulated by 7% polyethylene glycol (PEG6000) for 24 h. Electrolyte leakage, as well as proline and malondialdehyde (MDA) content, were subsequently measured. The results showed that all five *PuTPLs*-OE lines exhibited significantly lower electrolyte leakage and MDA content compared to the wild type (WT) under drought stress (*p* < 0.001), indicating reduced membrane damage and lipid peroxidation. Furthermore, proline content was significantly elevated in the *PuTPLs*-OE lines compared to the WT following drought treatment (*p* < 0.001) (Figure 10).

Notably, electrolyte leakage in the overexpression lines was reduced by 25–40% relative to the WT, while MDA levels decreased by 30–45%, demonstrating a substantial alleviation of oxidative stress. Concurrently, proline accumulation increased 1.8- to 2.3-fold in the transgenic lines, suggesting enhanced osmotic adjustment capacity. These physiological improvements were consistent across all five *PuTPLs*-OE lines genotypes, supporting functional conservation among members of this subfamily in drought adaptation. Together, these results demonstrate that *PuTPLs*-OE lines enhance drought tolerance by promoting proline accumulation, maintaining membrane integrity, and reducing oxidative damage under water deficit conditions.

## 3. Discussion

The TPL/TPR family is highly conserved across plant evolution, ranging from algae to angiosperms. The *A. thaliana* genome contains five *TPL/TPR* genes, including *TPL* and its four homologs *TPR1*–*TPR4* [31,32]. In contrast, the family has expanded to six members in tomato (*Solanum lycopersicum*) [33] and 18 members in *Brassica napus* [20], suggesting gene duplication and functional divergence during evolution. This study systematically identified 21 *PuTPLs*, which serve essential functions in transcriptional repression and drought stress responses. Phylogenetic analysis categorized these 21 *PuTPLs* into five subfamilies (*PuTPL*, *PuTPR1*, *PuTPR2*, *PuTPR3*, *PuTPR4*) demonstrating close relationships with their counterparts in Arabidopsis. The substantial expansion of the *PuTPLs*, compared to Arabidopsis and tomato, is accompanied by notable structural diversity despite overall conservation. This variation in family size reflects species-specific adaptations in transcriptional regulatory mechanisms, providing an evolutionary context for investigating *TPL/TPR* functions in non-model plants.

The TPL/TPR family represents a major class of transcriptional corepressors in plants, playing a central role in the negative regulation of gene expression. The N-terminal LisH and CTLH domains of these proteins’ mediate interactions with various transcriptional repressors. Unlike other members of the Groucho/Tup1 family, TPL/TPR proteins contain multiple WD40 repeat domains in their central regions, which facilitate not only binding to the EAR repression motif (LxLxL/DLNxxP) but also dimerization with proteins lacking a canonical EAR motif, such as WUSCHEL and HDACs. This enables their participation in diverse chromatin-modifying and transcriptional repression complexes [25,31]. Although the *PuTPLs* maintain structural conservation characterized by CTLH and WD40 domains, variations exist. For instance, four adjacent *PuTPR1* genes on chromosomes 11/12 display structural heterogeneity: *PuTPR1-1* and *PuTPR1-3* lack the LisH domain, whereas *PuTPR1-2* and *PuTPR1-4* contain dual LisH/CTLH domains (Figure 3B). These specific variations, likely resulting from segmental deletion and duplication during chromosomal replication, did not alter drought responsiveness (Figure 6B,C). A larger-scale deletion event was observed on chromosome 25, which is homologous to chromosome 26, resulting in the loss of the *PuTPR3-3* ortholog (Figure 4A). Collectively, these observations indicate that while *PuTPLs* maintain high evolutionary conservation, they also exhibit considerable genetic diversity. This diversity contrasts with their fundamental physicochemical properties. Analysis revealed striking similarities among TPL/TPR proteins in amino acid length, molecular weight, hydrophobicity, and secondary structure, which aligned closely with their orthologs in *A. thaliana* (Figure 2A,B). This remarkable conservation underscores strong evolutionary pressure maintaining these core features, likely due to their essential roles in transcriptional repression. The minimal divergence in protein properties supports the hypothesis that neofunctionalization in this gene family occurs primarily through changes in regulatory elements rather than protein coding sequences [34,35]. This high degree of conservation parallels observations in other plants, suggesting that the TPL/TPR-mediated repression mechanism represents an ancient and vital regulatory pathway in plant development and stress responses.

The *TPL/TPR* genes play a central role in various stress responses, including drought and temperature extremes, and regulating multiple hormonal pathways, such as jasmonic acid (JA), auxin, and salicylic acid (SA) signaling [36,37,38]. Our bioinformatic analysis of promoter *cis*-regulatory elements revealed that *PuTPLs* contain numerous stress- and hormone-responsive motifs. Our motif enrichment analysis revealed a striking overrepresentation of drought and abiotic stress-responsive *cis*-regulatory elements within the promoter regions of the target gene set. The most significantly enriched motifs were predominantly bound by transcription factors from the *AP2/ERF* superfamily (including *DREB2D*, *DREB2G*, *ERF5*, *ERF014*, and *ERF057*), which are well-established master regulators of dehydration, salinity, and cold stress responses. The equally significant enrichment of the *ABF4* binding motif, a central component of the abscisic acid (ABA) signaling pathway, further underscores the critical role of ABA-mediated transcriptional regulation in the drought stress response. Additionally, the identification of enriched motifs for *NAC* family TFs (*NAC025*, *NAC057*) reinforces the network of stress-responsive regulation. The co-enrichment of these specific TF binding motifs demonstrates a coordinated transcriptional regulatory code, suggesting that the expression of the target genes is likely governed by a synergistic interplay between ethylene, ABA, and other stress signaling pathways to orchestrate adaptive physiological outcomes under drought conditions (Figure 3C) [39,40,41]. This architectural feature of the promoter regions is consistent with the known role of TPL/TPR proteins as transcriptional corepressors that recruit a wide range of transcription factors and chromatin-modifying complexes. Thus, the *cis*-element landscape not only corroborates the functional relevance of *PuTPLs* in crosstalk between signaling cascades but also provides a mechanistic basis for their context-specific regulatory capacity in stress adaptation and hormonal integration.

The EAR motif (LxLxL or DLNxxP) represents the most prevalent repression domain in plant transcription factors. It mediates transcriptional repression by recruiting corepressors such as TPL/TPR proteins, leading to the formation of multiprotein repressor complexes. These complexes can directly bind to promoter regions of target genes and/or recruit chromatin modifiers to reduce histone acetylation, thereby suppressing transcription [25,26]. The high evolutionary conservation of the EAR motif across plant species and transcription factor families underscores its critical role in regulating genes involved in development, stress responses, and hormone signaling pathways. This repression mechanism allows plants to rapidly modulate gene expression in response to dynamic environmental conditions [31]. Our interaction network analysis revealed two distinct classes of TPL/TPR-interacting partners: (1) EAR motif-dependent interactors, which exclusively contain canonical LxLxL (or DLNxxP)-type EAR motifs and represent known corepressor recruitment targets—such as PuCSLD3, PuNUP107, and members of the PuSMXL family—and (2) EAR motif-independent interactors, including proteins with alternative repression domains like the TPL/TPR homologs LUG and LUH. This classification aligns with previously established genetic interactions [42,43,44,45,46,47]. Both interaction modes are operational in *PuTPLs*. Notably, the evolutionarily conserved EAR-mediated repression mechanism functions independently of transcription factors. We observed variations in the strength of interactions between different types of proteins and TPL/TPR proteins (Figure 10), which may be attributed to differences in their conformational properties. Many non-transcription factor proteins containing the EAR motif, such as the adaptor protein NINJA, serve as cores of large macromolecular complexes and require association with multiple partner proteins to adopt a functionally appropriate conformation [23]. In simplified artificial systems like the yeast two-hybrid (Y2H) assay, the absence of these auxiliary components may lead to partial occlusion of the EAR motif or structural instability, resulting in weaker observed interactions with TPL proteins. In contrast, transcription factors generally possess more independent structural domains, and their EAR motifs are likely more accessible, facilitating stronger detectable interactions in experimental settings. Throughout evolution, transcription factors may have optimized the structural environment around the EAR motif to maximize its exposure to TPL/TPR proteins, enabling efficient recruitment for direct transcriptional repression of downstream genes. On the other hand, the conformational properties of the EAR motif in non-transcription factors might not be optimized for maximal binding affinity, but rather to support dynamic functional requirements within their respective complexes. To refine and expand these predicted networks, we propose a systematic experimental workflow comprising: (1) comprehensive yeast two-hybrid library screening to complete the interaction landscape; followed by (2) extensive validation of key interactions using bimolecular fluorescence complementation (BiFC) and co-immunoprecipitation (Co-IP), coupled with functional characterization of EAR motif variants.

Current studies have established that drought stress triggers substantial transcriptional activation of diverse gene families, including zinc finger proteins [4], ethylene-responsive factors [10], and auxin signaling components [11]. The root-specific upregulation correlates with morphological adaptations including primary root elongation and lateral root suppression [6,48], while leaf induction coincides with stomatal regulation genes [49], indicating tissue-specific roles in drought avoidance strategies [1]. The *PuTPLs* demonstrated distinct organ-specific expression patterns under drought conditions, exhibiting differential regulation between leaves and roots (Figure 5, Figure 6, Figure 7, Figure 8 and Figure 9). This spatial divergence may be linked to the enrichment of stress-related transcription factors with potential light-responsive properties, such as *bHLH78* and *ABF4*, identified in their promoter regions (Figure 3C). This suggests an evolutionarily conserved mechanism for partitioning drought responses between aboveground and underground plant organs. It is plausible that the activity of these TFs integrates light and drought signaling pathways to fine-tune tissue-specific repression of growth-related genes and facilitate resource reallocation under water deficit [50]. Our temporal expression analysis revealed distinct peaks during early and intermediate drought phases. To validate these patterns and establish their correlation with stress intensity, we will utilize stable transgenic overexpression lines. Three-month-old plants will be subjected to controlled drought stress regimens at three severity levels: mild (SRWC > 60%), moderate (40–60%), and severe (<40%). This systematic approach will elucidate the *PuTPLs* drought response mechanisms. The combination of temporal and organ-specific expression dynamics suggests that *PuTPLs* may serve as integrative hubs in drought response networks, linking environmental cues with developmental reprogramming. Further investigation into protein–protein interactions and target gene repression under different drought regimes will be essential to unravel the molecular mechanisms underlying this spatial-temporal functional divergence.

The observed physiological responses in *PuTPLs*-OE lines under drought stress provide evidence supporting the functional role of these corepressors in enhancing drought tolerance. The significantly reduced electrolyte leakage and MDA content indicate that overexpression of *PuTPLs* effectively stabilizes membrane integrity and reduces oxidative damage, likely through the regulation of stress-responsive genes involved in reactive oxygen species (ROS) scavenging and lipid protection. Concurrent elevation in proline levels suggests that *PuTPLs* may modulate osmotic adjustment mechanisms, possibly by suppressing negative regulators of proline biosynthesis or promoting the expression of proline synthetic enzymes [51]. These findings align with the known role of TPL/TPR proteins in recruiting chromatin-modifying complexes to repress gene expression and imply that *PuTPLs* contribute to drought adaptation by fine-tuning both osmotic balance and oxidative stress responses [52]. Further investigation is needed to identify the direct target genes of *PuTPLs* and elucidate the epigenetic mechanisms through which they confer these advantageous physiological traits.

## 4. Materials and Methods

### 4.1. Identification and Characterization

The *P*. *ussuriensis* genome project has been deposited with the NCBI under the BioProject number SUB13708257. The whole-genome sequencing data were deposited in the sequence Read Archive (SRA) under accession number PRJNA998551. The *P*. *ussuriensis* genome assembly has been deposited in Figshare (https://doi.org/10.6084/m9.figshare.24013941.v3 accessed on 3 April 2025). The genome data of *A. thaliana* and *P. trichocarpa* were downloaded from the Phytozome13 (https://phytozome-next.jgi.doe.gov/ accessed on 11 April 2025) [53]. The Fasta Extract tool in TBtools-II was employed to screen for *TPL/TPR* genes in the *P. ussuriensis* gene database [54]. Subsequently, homologous *TPL/TPR* genes in *A. thaliana* and *P. trichocarpa* were identified using BLASTN analysis (score value ≥ 100, e-value ≤ 1 × 10^−10^).

### 4.2. Physicochemical Characterization and Secondary Structure Prediction

Physicochemical properties (theoretical pI, molecular weight, number of amino acids, stability index, aliphatic index and grand average of hydropathicity) were predicted using Expasy ProtParam (https://www.expasy.org/ accessed on 14 April 2025) [55]. Secondary structure prediction was performed using NPS@ (https://npsa.lyon.inserm.fr/cgi-bin/npsa_automat.pl?page=/NPSA/npsa_dsc.html accessed on 19 April 2025) [56]. All protein physicochemical properties were normalized using min-max scaling, whereby values were linearly scaled to the [0, 1] interval to remove unit-based variations and facilitate enhanced visualization comparability among multiple dimensions. Data visualization was performed using MATLAB R2023a (https://www.mathworks.com/products/matlab.html accessed on 24 April 2025).

### 4.3. Phylogeny Reconstruction and Multiple Sequence Alignment

The Muscle algorithm with default parameter was employed for multiple sequence alignment. The evolutionary relationships of the *PuTPLs* were inferred using MEGA 12 with the neighbor-joining (NJ) method [57,58,59].

### 4.4. Analysis of Conserved Domains, Cis-Regulatory Elements and Gene Structure

Conserved domains were predicted using the SMART online tool (https://smart.embl.de/ accessed on 17 May 2025) [60,61,62,63]. Gene structures were visualized using TBtools-II [54]. *Cis*-regulatory elements within the promoter region were screened using the JASPAR database (https://jaspar.elixir.no/ accessed on 17 May 2025) [64], followed by enrichment analysis of 3000 base pairs upstream of the start codon utilizing AME (https://web.mit.edu/meme_v4.11.4/share/doc/overview.html accessed on 17 May 2025) [65], with visualization performed using MATLAB.

### 4.5. Chromosomal Localization and Collinear Relationship

*TPL/TPR* genes were mapped to *P. ussuriensis* chromosomes using the Gene Location Visualize tool in TBtools-II [54]. Intra-genomic collinearity was analyzed using MCScanX and visualized with Circos tools in TBtools-II. Inter-genomic collinear relationships among *P. ussuriensis*, *A. thaliana*, and *P. trichocarpa* were illustrated using the Dual Systeny Plot in TBtools-II.

### 4.6. Plant Materials and Stress Treatment

Wild-type *P. ussuriensis* plants were maintained through successive subculture in our laboratory. Plants were cultivated on 1/2 MS medium at 25 °C under a 16 h light/8 h dark photoperiod (light intensity of 46 μmol m^−2^·s^−1^) [4]. Three-week-old tissue-cultured seedlings were used for stress treatments and RNA extraction. For drought stress, 7% (*w*/*v*) PEG 6000 was incorporated into the 1/2 MS solid medium. Wild-type plantlets (1-week-old, with visible root primordia) were transferred to stress medium. The stress treatment duration was recorded consecutively for 7 days, starting from the time of transfer.

### 4.7. Total RNA Extraction, Reverse Transcription, and RT-qPCR Analysis

Total RNA was extracted from roots and leaves using the CTAB method [66]. RNA quality was verified by agarose gel electrophoresis. Qualified samples were reverse-transcribed using reagents from Vazyme Biotech Co., Ltd (Nanjing, China). Quantitative RT-qPCR was performed using the SYBR Green method (Vazyme Biotech Co., Ltd, Nanjing, China) with three technical replicates per biological sample. The primers used are listed in Supplementary Table S3.

### 4.8. Interaction Network Prediction

Interaction networks were predicted using the STRING database (https://cn.string-db.org/ accessed on 20 May 2025) [67]. Interactions were filtered with a medium-confidence threshold (score ≥ 0.400). The top 10 interactors based on confidence scores were visualized. Target protein sequences were obtained from UniProt (https://www.uniprot.org/database/DB-0028 accessed on 20 May 2025) [68]. Potential interacting genes in *P. ussuriensis* were identified via TBlastN (score value ≥ 100, e-value ≤ 1 × 10^−10^) searches. EAR motifs (LxLxL/DLNxxP) were identified among these candidates [47]. Networks were visualized using Cytoscape [69].

### 4.9. Cloning and Vector Construction

The full-length CDSs of *PuTPL-2*, *PuTPR1-1*, *PuTPR2-2*, *PuTPR3-2*, and *PuTPR4-2* were amplified by PCR using KOD DNA Polymerase (TOYOBO Osaka, Japan) with primers listed in Supplementary Table S3. PCR products were purified and cloned into pGBKT7 (bait) and pBI121 (overexpression) vectors using the SmaI restriction site via homologous recombination (ClonExpress^®^ II One Step Cloning Kit, Vazyme). For Y2H, CDSs of putative interactors (*PuNUP107* and *PuSMXL6*) were cloned into the pGADT7 (prey) vector. All constructs were transformed into *E. coli* DH5α competent cells (Rabbit) and verified by Sanger sequencing. Forward and reverse primers containing the designed nucleotide substitutions were used to amplify the target genes in two separate PCR reactions, and the resulting fragments were fused into complete mutated coding sequences (CDS) via overlap PCR. The final products were cloned into the pGADT7 vector and confirmed by Sanger sequencing to ensure the accuracy of the introduced mutations. The primers used are listed in Supplementary Table S3.

### 4.10. Yeast Two-Hybrid Assays

Bait and prey plasmids were co-transformed into *S. cerevisiae* Y2HGold strain using the PEG/LiAc method [70], with competent cells prepared using the PYEAST Kit (Xianyang, China). Transformants were selected on SD/-Trp-Leu (SD/-TL) and SD/-Trp-Leu-His-Ade (SD/-TLHA) media (Coolaber). X-α-Gal (0.1 M) was added to SD/-TLHA for blue/white screening. pGBKT7-LAM/pGADT7 and pGBKT7-p53/pGADT7 served as negative and positive controls, respectively. Plates were incubated at 28 °C for 5 days, and interactions were assessed based on blue colony formation. Three biological replicates were performed.

### 4.11. Transient Expression and Physiological Index Analysis

Recombinant plasmids were introduced into *A. tumefaciens* GV3101 by the freeze–thaw method and cultured to OD_600_ = 0.6. Three-week-old wild-type seedlings were immersed in hypertonic solution (25% sucrose in 1/2 MS, pH 5.8) for 2 h, then in transformation solution (1/2 MS, 150 μM acetosyringone, 2.5% sucrose, 0.01% Tween-20, agrobacterial suspension, pH 5.8) at 25 °C with shaking (120 rpm) for 6 h [71]. After washing, seedlings were plated on 1/2 MS medium for 48 h dark incubation. Five independent overexpression lines were treated with 7% PEG6000. Electrolyte leakage was measured using a conductivity meter. Proline and MDA contents were determined using commercial assay kits (Jiancheng Bioengineering Institute Nanjing, China).

### 4.12. Statistical Analysis

Data from three biological replicates were presented as mean ± SD. Statistical significance (*p* < 0.05) was determined by one-way ANOVA followed by Tukey’s test using Origin 2024 software (https://www.originlab.com/ accessed on 29 May 2025).

## 5. Conclusions

In this study, we identified and characterized 21 *PuTPLs*, revealing their evolutionary conservation and involvement in drought stress responses. Phylogenetic analysis classified these genes into five subfamilies distributed across 12 chromosomes, demonstrating high evolutionary conservation with *A. thaliana* and *P. trichocarpa*. Functional investigations showed that most *PuTPLs* are upregulated under drought stress, with their responses varying across subfamilies, suggesting functional diversification. Transient overexpression of selected *PuTPLs* significantly enhanced drought tolerance, supported by reduced electrolyte leakage and malondialdehyde levels, along with increased proline accumulation. Interaction network analysis further indicated that *PuTPLs* associate with key stress-responsive regulators and epigenetic modifiers, highlighting their potential role in repressive signaling networks.

These findings underscore the importance of TPL/TPR-mediated transcriptional repression as a regulatory mechanism in drought resistance. This study provides a foundation for further functional analysis of this gene family and offers promising targets for improving drought tolerance in woody plants through genetic engineering.

## Data Availability

All data are contained within the article and the Supplementary Materials.

## Supplementary Materials

The following supporting information can be downloaded at: https://www.mdpi.com/article/10.3390/plants14203213/s1, Supplementary Table S1. Physicochemical property analysis of PuTPL/TPR proteins. Supplementary Table S2. Prediction of secondary structures in PuTPL/TPR proteins. Supplementary Table S3. Primers used in this study. Supplementary Figure S1. Gene structure and conserved domains analysis. Supplementary Figure S2. Chromosomal distribution of *PuTPLs*. Supplementary Figure S3. Expression analysis of *PuTPR1-5* to *PuTPR1-8* under drought stress. Supplementary Figure S4. Phylogenetic analysis of *TPL* family genes in *P. ussuriensis*, *A. thaliana*, and *P. trichocarpa*. Supplementary Figure S5. Phylogenetic analysis of *TPR1* family genes in *P. ussuriensis*, *A. thaliana*, and *P. trichocarpa.* Supplementary Figure S6. Phylogenetic analysis of *TPR2* family genes in *P. ussuriensis*, *A. thaliana*, and *P. trichocarpa*. Supplementary Figure S7. Phylogenetic analysis of *TPR3* family genes in *P. ussuriensis*, *A. thaliana*, and *P. trichocarpa*. Supplementary Figure S8. Phylogenetic analysis of *TPR3* family genes in *P. ussuriensis*, *A. thaliana*, and *P. trichocarpa*.
