# Supplementary material for: Genome-Wide Identification, Drought-Responsive Expression, and EAR-Mediated Regulatory Network Construction of TOPLESS Genes in Populus ussuriensis Kom
Source: Plants (Basel). 2025 Oct 19;14(20):3213. doi: 10.3390/plants14203213 (PMC12566939; doi:10.3390/plants14203213)
Supplement: Supplementary file 1 [file plants-14-03213-s001.zip › Supplementary Data.pdf]

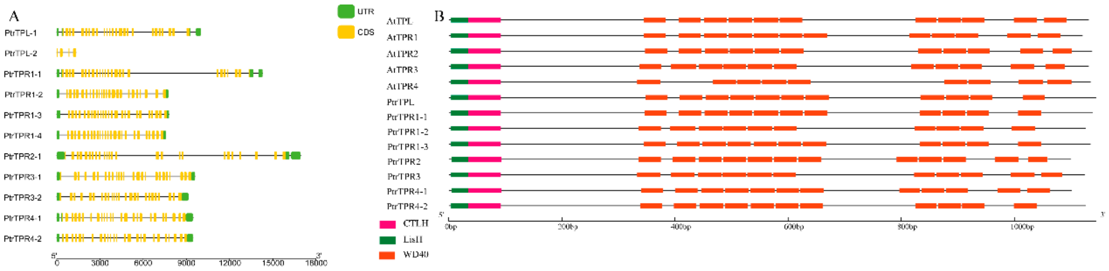

Supplementary Figure S1. Gene structure and conserved domains analysis (A) Gene structure of *Populus trichocarpa TPL/TPR* genes. Untranslated regions (UTRs) are shown in green, coding sequences (CDS) in yellow, and introns are represented by gray lines. (B) Conserved domain architectures of TPL/TPR proteins in *Arabidopsis thaliana* and *P. trichocarpa*. Specific domains are color-coded: CTLH (pink), LisH (green), and WD40 repeats (orange).

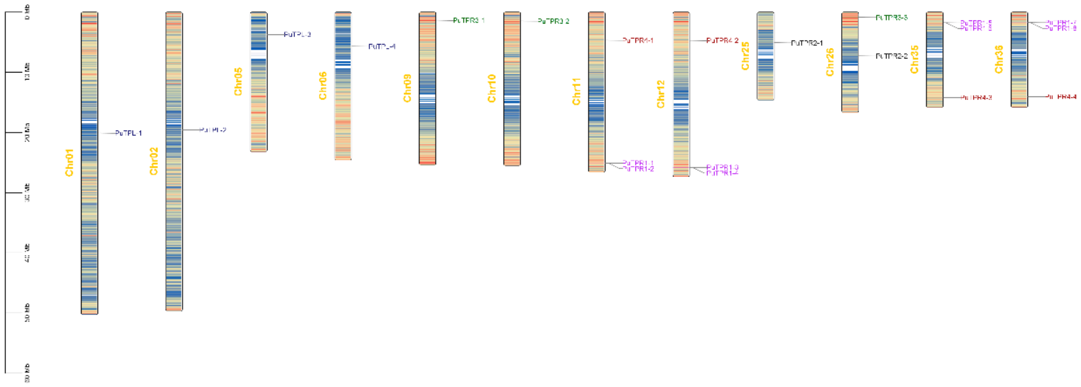

Supplementary Figure S2. Chromosomal distribution of *PuTPLs*. Chromosome names are labeled on the left. The gray background lines within chromosomes represent gene density. The locations of *PuTPLs* are indicated by colored bars.

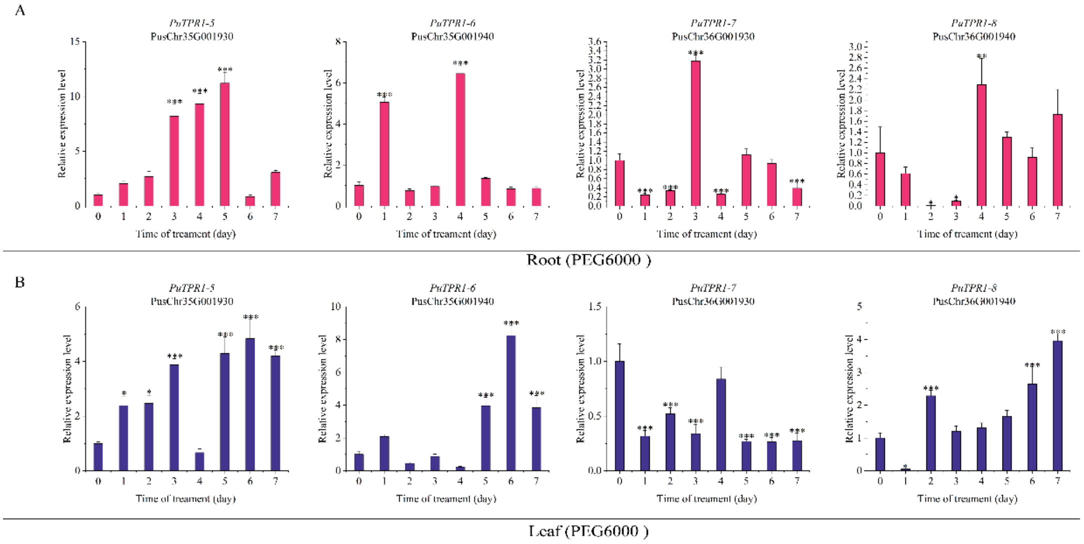

Supplementary Figure S3. Expression analysis of *PuTPR1-5* to *PuTPR1-8* under drought stress. (A) Relative expression levels of *PuTPR1* in roots at different time points under drought stress. (B) Relative expression levels of *PuTPR1* in leaves at different time points under drought stress. For (A) and (B): Gene expression was normalized to *PuActin* used as an internal reference. Data are presented as the mean ± SD (n = 3 biological replicates). Statistical significance was determined by one-way ANOVA (Tukey's test): *$p \leqslant 0.05$, **$p \leqslant 0.01$, ***$p \leqslant 0.001$.

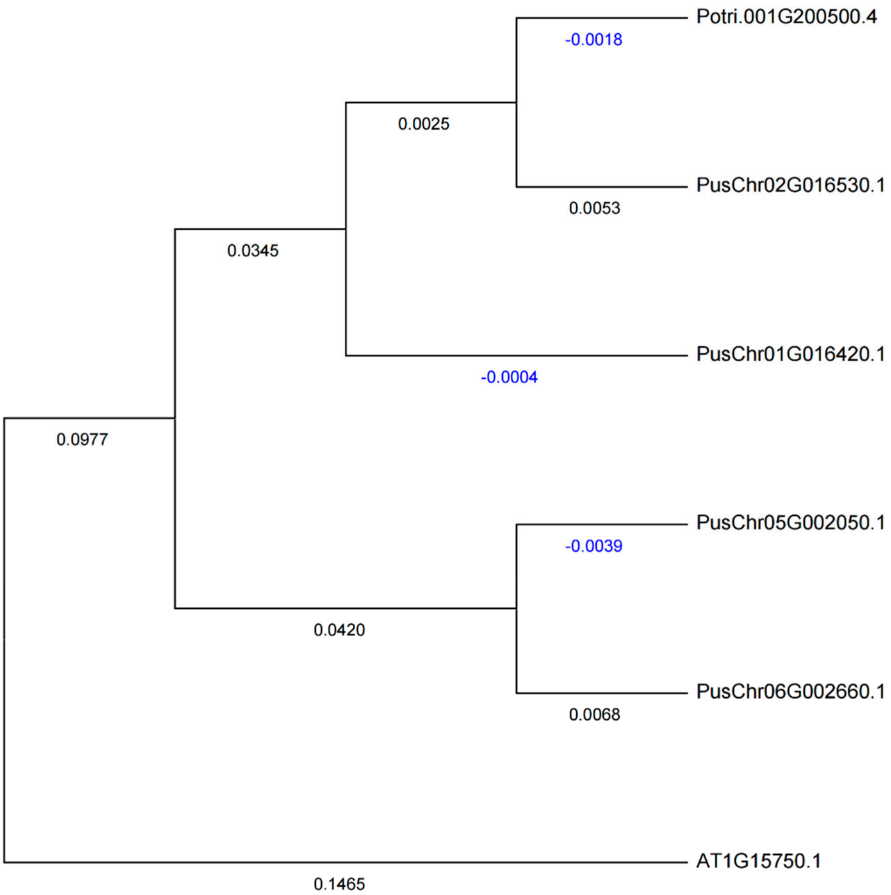

Supplementary Figure S4. Phylogenetic analysis of *TPL* genes in *P. ussuriensis*, *A. thaliana*, and *P. trichocarpa*.

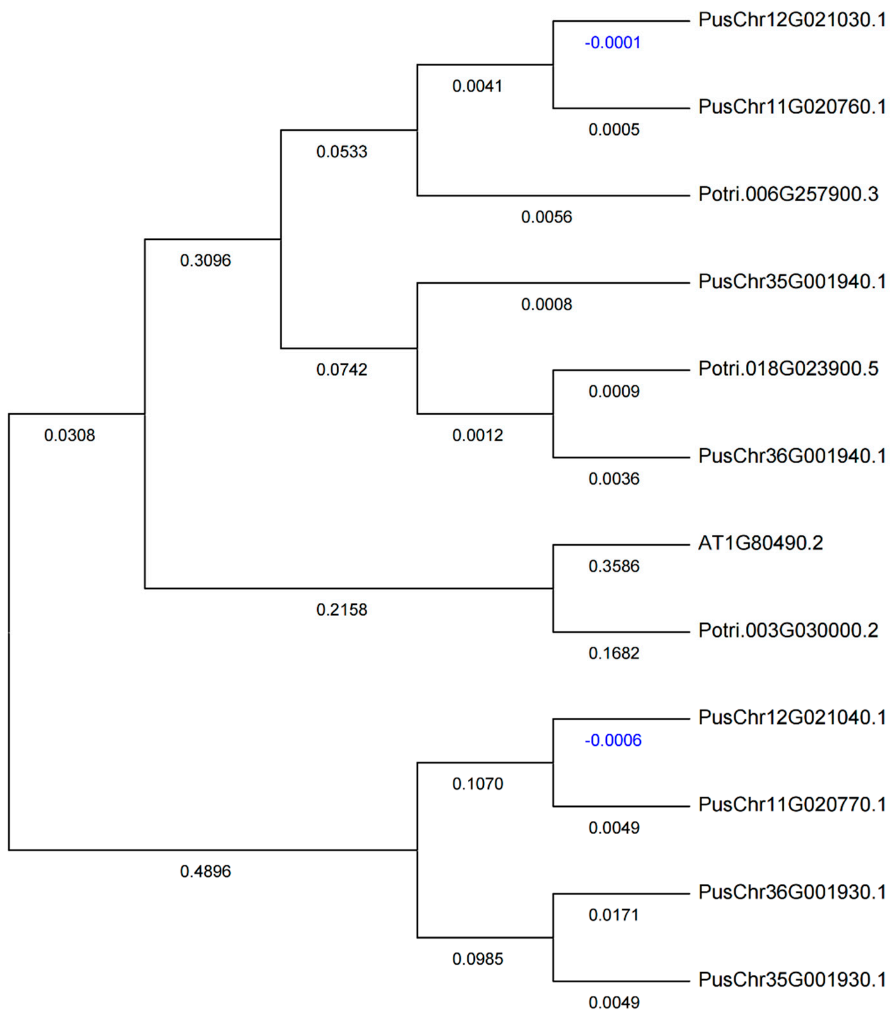

Supplementary Figure S5. Phylogenetic analysis of *TPR1* genes in *P. ussuriensis*, *A. thaliana*, and *P. trichocarpa*.

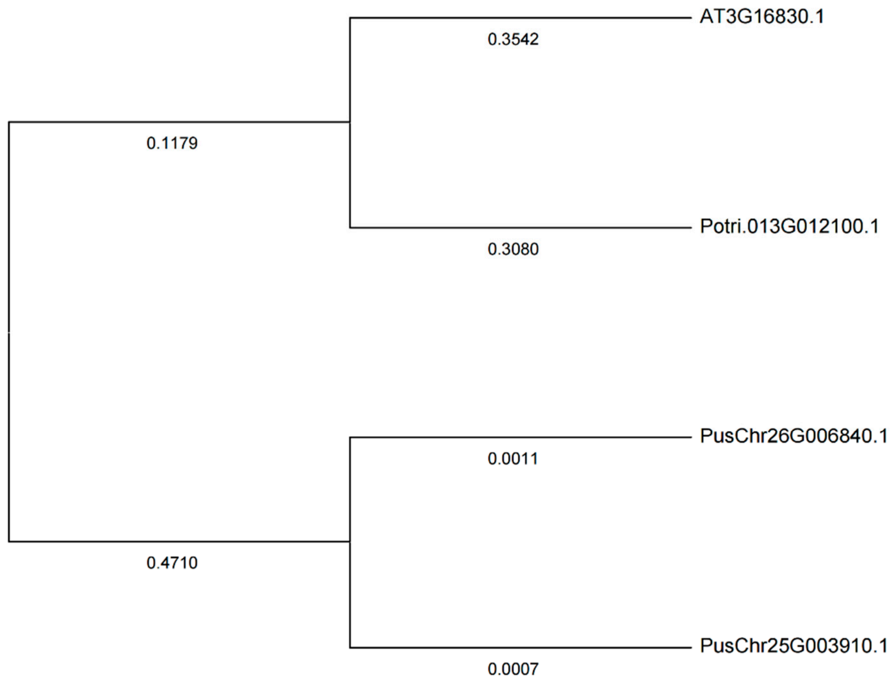

Supplementary Figure S6. Phylogenetic analysis of *TPR2* genes in *P. ussuriensis*, *A. thaliana*, and *P. trichocarpa*.

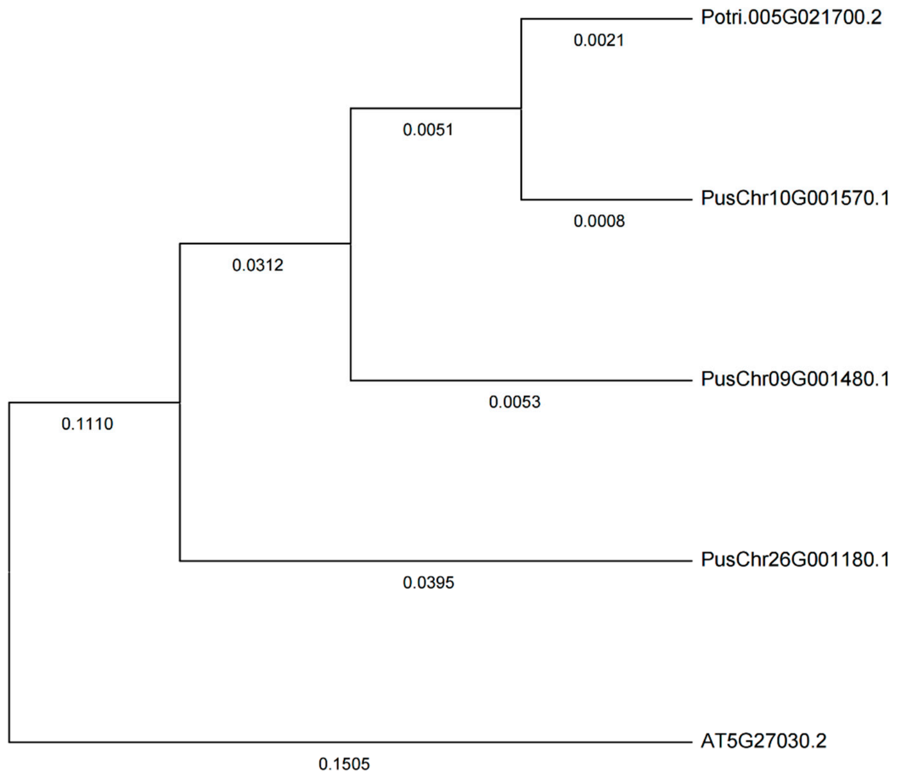

Supplementary Figure S7. Phylogenetic analysis of *TPR3* genes in *P. ussuriensis*, *A. thaliana*, and *P. trichocarpa*.

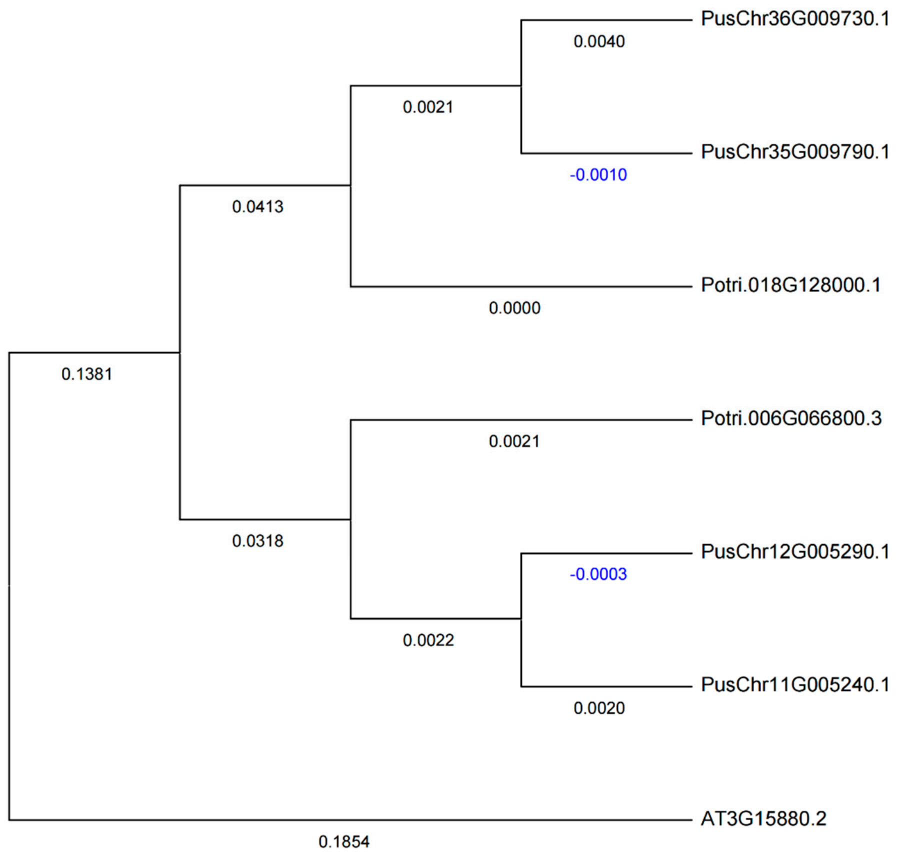

Supplementary Figure S8. Phylogenetic analysis of *TPR4* genes in *P. ussuriensis*, *A. thaliana*, and *P. trichocarpa*.
