# Supplementary material for: Genome-Wide Identification, Drought-Responsive Expression, and EAR-Mediated Regulatory Network Construction of TOPLESS Genes in Populus ussuriensis Kom
Source: Plants (Basel). 2025 Oct 19;14(20):3213. doi: 10.3390/plants14203213 (PMC12566939; doi:10.3390/plants14203213)
Supplement: Supplementary file 1 [file plants-14-03213-s001.zip › Supplementary table.pdf]

Supplementary TableS1. Physicochemical property analysis of PuTPL/TPR proteins.

Gene ID	Sequence ID	Number of Amino Acids	Molecular weight (Da)	Theoretical pI	Instability index	Aliphatic index	Grand average of hydropathicity
TPL-1	PusChr01G016420	1143	125549.39	6.63	39.65	82.27	-0.251
TPL-2	PusChr02G016530	1235	136206.79	7.95	39.83	83	-0.263
TPL-3	PusChr05G002050	1137	124905.6	6.61	40.3	81.84	-0.273
TPL-4	PusChr06G002660	1211	132803.76	6.93	40.47	83.29	-0.227
TPR1-1	PusChr11G020760	1087	119813.18	6.71	36.44	82.91	-0.26
TPR1-2	PusChr11G020770	1285	142361.7	5.49	40.77	82.66	-0.289
TPR1-3	PusChr12G021030	1087	119813.18	6.71	36.44	82.91	-0.26
TPR1-4	PusChr12G021040	1282	142040.54	5.49	40.6	83.99	-0.264
TPR1-5	PusChr35G001930	1085	120349.21	5.3	41.19	86.13	-0.238
TPR1-6	PusChr35G001940	1118	123384.63	6.47	40.84	84.02	-0.225
TPR1-7	PusChr36G001930	1154	128716.71	5.28	42.53	85.79	-0.242
TPR1-8	PusChr36G001940	1130	124630.01	6.55	40.48	84.42	-0.229
TPR2-1	PusChr25G003910	1136	125154.15	6.63	42.7	81.07	-0.302
TPR2-2	PusChr26G006840	1135	125040.04	6.63	42.94	81.14	-0.299
TPR3-1	PusChr09G001480	963	106871.86	8.08	36.57	83.36	-0.246
TPR3-2	PusChr10G001570	1125	124430.38	7.13	37.88	83.06	-0.277
TPR3-3	PusChr26G001180	1124	124215.04	6.91	37.09	83.31	-0.283
TPR4-1	PusChr11G005240	1224	135828.02	8.6	42.15	78.83	-0.347
TPR4-2	PusChr12G005290	1131	124678.11	6.57	40.95	80.14	-0.323
TPR4-3	PusChr35G009790	1125	123920.25	6.75	39.21	79.88	-0.323
TPR4-4	PusChr36G009730	1170	129035.38	6.86	38.71	80.32	-0.313

Supplementary Table S2. Prediction of secondary structures in PuTPL/TPR proteins.

Gene ID	Sequence ID	Fraction of alpha helices (%)	Fraction of extended strand (%)	Fraction of random coil (%)
TPL-1	PusChr01G016420	17.94	15.84	66.23
TPL-2	PusChr02G016530	18.79	13.77	67.45
TPL-3	PusChr05G002050	20.84	13.46	65.7
TPL-4	PusChr06G002660	18.33	13.96	67.71
TPR1-1	PusChr11G020760	15.73	17.57	66.7
TPR1-2	PusChr11G020770	24.98	15.33	59.69
TPR1-3	PusChr12G021030	15.73	17.57	66.7
TPR1-4	PusChr12G021040	21.29	17.86	60.84
TPR1-5	PusChr35G001930	17.42	18.8	63.78
TPR1-6	PusChr35G001940	17.17	16.01	66.82
TPR1-7	PusChr36G001930	20.62	16.46	62.91
TPR1-8	PusChr36G001940	15.22	16.46	68.32
TPR2-1	PusChr25G003910	20.16	14	65.85
TPR2-2	PusChr26G006840	19.47	15.42	65.11
TPR3-1	PusChr09G001480	17.76	18.07	64.17
TPR3-2	PusChr10G001570	16	16.89	67.11
TPR3-3	PusChr26G001180	15.84	16.73	67.44
TPR4-1	PusChr11G005240	18.95	14.62	66.42
TPR4-2	PusChr12G005290	19.27	14.94	65.78
TPR4-3	PusChr35G009790	19.11	15.91	64.98
TPR4-4	PusChr36G009730	19.57	13.08	67.35

Supplementary Table S3. Primers used in this study

Genes and primers used in RT-qPCR Analyses		
Genes	Primers	Sequences (5'-3')
TPL-1	forward	GATGGGGATTCATTCATTG
	reverse	GTTGAAGCGGATACGTGGGC
TPL-2	forward	ATGCTGAGTCACCACCTAAGC
	reverse	CAAGCTCTCGACTGAGAGAAGA
TPL-3	forward	ATTGATGCTGATGGAGGCCT
	reverse	CTGCTAGTTGCAGCTGCTGCTG
TPL-4	forward	ATGCTCAGAATTGAAACGGCG
	reverse	GTCGGCATGAGATGAGTG
TPR1-1	forward	CCGTGACAAATTGCAGTATCC
	reverse	GGAATGACCCATTCGCAAC
TPR1-2	forward	GGCCAGATGGTGTATGCTAGC
	reverse	CTGATTAGCTTCGGATTCAGC
TPR1-3	forward	AGTTGCAAATGGGTCATTCC
	reverse	CCTGTCAACATTACCCTGTCC
TPR1-4	forward	AATGACTAGGGACGTCACC
	reverse	CCTTGTTGTCCAATGCCTCCAG
TPR1-5	forward	CTGGATGGACTTGTCTC
	reverse	ATAATTAGCCTCGGATTCGGC
TPR1-6	forward	CCAGCACCAGCTCCAATACC
	reverse	GTCTTGGGCAACTCTTCGGTC
TPR1-7	forward	ATGGGATGCCGAGTGTTTTGG
	reverse	GTCGAGTATCTGTTGTCATC
TPR1-8	forward	ATGCAGGCAAGGCAGATACAC
	reverse	CTTCTCAACCTCATCCCAGTC
TPR2-1	forward	CTGTTGGTTGTCCATGAAAGCC
	reverse	ATAGGCAGTGCTGCCTGCAGGG
TPR2-2	forward	GTTGGTTGTCCATGAAAGCC
	reverse	CCAAAGGATAGGCAGTGC
TPR3-1	forward	ATGTCAGCCACTGGTGGGAAGG
	reverse	CAAACAGCATTGCTCCTATG
TPR3-2	forward	ATTGCCCCCTCAGTATACAAC
	reverse	TCTTTGCAACTGATCTGGC
TPR3-3	forward	TGCTGGTGCCTTAGCTGGTTGG
	reverse	GCCTGTTGTCTAGATGCTGG
TPR4-1	forward	GAGCTTGTTCAGAACCAGC
	reverse	CTATGGAGAAGGGTCGGAG
TPR4-2	forward	GCAGTAGCACTCTTAAAGC

	reverse	CCATACTCTTGACAGCAG
TPR4-3	forward	CGTATACTCCTACCATGGCGGT
	reverse	CTTTCCATCAGTTGCGGTTG
TPR4-4	forward	AACAGACGGTGGAATTCATGTGC
	reverse	CACCGTCGGTCAACATGAACATC
Actin	forward	TGTTGCCCTTGACTATGAGCAGGA
	reverse	ACGGAATCTCTCAGCTCCAATGGT
**Genes and primers used in cloning**		
TPL-2	forward	ATGCTGAGTCACCACCTAAGC
	reverse	TCTCTGAGCTTGATCTGAACCT
TPR1-1	forward	ATGATTCCCTTTTGTTTC
	reverse	CTCCATGTTCTGGGGTTGATC
TPR2-2	forward	ATGTCTTCCTTAAGCAGGGAAC
	reverse	CCTGGAAGGGAGCTCTGACAGGTGA
TPR3-2	forward	ATGTCGTCTTTGAGTAGAGAATTGGTG
	reverse	TCTTTGCAACTGATCTGGC
TPR4-2	forward	ATGTCTTCGCTTAGCCGAGAGCTTG
	reverse	CCTTTGAGCTTGTTCAGAACCA
NUP107	forward	ATGGAGGATGTTGAAATGGATGTG
	reverse	AGATGCAAGTTCCTCCTGAAGCTCCAT
	A- forward	CACGCCGTGGCCGTGGCCAGGTATTTACATGCTGAGGAAATGCAGGA
	A- reverse	CCTGGCCACGGCCACGGCGTGAGCACCAAAACGAATCATCT
SMXL6	forward	ATGCCGACGCCGGTAGGCGTAGCGA
	reverse	CGGATTTATTCTTGAAGGCAGGCATATTCCA
	A- forward	TATGCCGATGCCAATGCCCCTGTAGAAGATACAGGAGAGT
	A- reverse	AGGGGCATTGGCATCGGCATAGGATCGCAATGCCTTATGG
**Genes and primers used in yeast two-hybrid assays**		
TPL-2	TPL-BD-F	ATGGAGGCCGAATTACCCGGGATGCTGAGTCACCACC
	TPL-BD-R	GCAGGTCGACGGATCCCCGGGTCTCTGAGCTTGATCTGAA
TPR1-1	TPR1-BD-F	ATGGAGGCCGAATTACCCGGGATGATTCCCTTTTGTTTC
	TPR1-BD-R	TGCAGGTCGACGGATCCCCGGGCTCCATGTTCTGGGGTTG
TPR2-2	TPR2-BD-F	ATGGAGGCCGAATTACCCGGGATGTCTTCCTTAAGCAGGG
	TPR2-BD-R	TGCAGGTCGACGGATCCCCGGGCCTGGAAGGGAGCTCTGA
TPR3-2	TPR3-BD-F	ATGGAGGCCGAATTACCCGGGATGTCGTCTTTGAGTAG
	TPR3-BD-R	TGCAGGTCGACGGATCCCCGGGTCTTTGCAACTGATCTGGC
TPR4-2	TPR4-BD-F	ATGGAGGCCGAATTACCCGGGATGTCTTCGCTTAGCCGAGA
	TPR4-BD-R	TGCAGGTCGACGGATCCCCGGGCCTTTGAGCTTGTTCAGAA
NUP107	NUP107-ADRec-F	GAGTGGCCATTATGGCCCGGGATGGAGGATGTTGAAATGGATGTG
	NUP107-ADRec-R	GCCGACATGTTTTTTCCCGGGAGATGCAAGTTCCTCCTGAAGCTCCAT
SMXL6	SMXL6-ADRec-F	GAGTGGCCATTATGGCCCGGGATGCCGACGCCGGTAGGCGTA
	SMXL6-ADRec-R	GCCGACATGTTTTTTCCCGGGCGGATTTATTCTTGAAGGCAGG
**Genes and primers used in transient expression**		
TPL-2	TPL-GFP-F	CGGGGGACTCTAGACTGGTACCCGGGATGCTGAGTCACCACCT
	TPL-GFP-R	ACTAGTCAGTCGACCCGGGAATCTCTGAGCTTGATCTGAACCT

TPR1-1	TPR1-GFP-F	CGGGGGACTCTAGACTGGTACCCGGGATGATTCCCTTTTGTTTC
	TPR1-GFP-R	ACTAGTCAGTCGACCCGGGAACTCCATGTTCTGGGGTTGATC
TPR2-2	TPR2-GFP-F	CGGGGGACTCTAGACTGGTACCCGGGATGTCTTCCTTAAGCAGG
	TPR2-GFP-R	ACTAGTCAGTCGACCCGGGAACCTGGAAGGGAGCTCTGACAGG
TPR3-2	TPR3-GFP-F	CGGGGGACTCTAGACTGGTACCCGGGATGTCGTCTTTGAGTAGA
	TPR3-GFP-R	ACTAGTCAGTCGACCCGGGAATCTTTGCAACTGATCTGGC
TPR4-2	TPR4-GFP-F	CGGGGGACTCTAGACTGGTACCCGGGATGTCTTCGCTTAGCCGA
	TPR4-GFP-R	ACTAGTCAGTCGACCCGGGAACCTTTGAGCTTGTTCAGAACCA
